# A Case of Syphilis with High Bone Arsenic Concentration from Early Modern Cemetery (Wroclaw, Poland)

**DOI:** 10.1515/biol-2019-0048

**Published:** 2019-11-17

**Authors:** Pawel Dabrowski, Michal Jerzy Kulus, Agata Cieslik, Zygmunt Domagala, Rafał J. Wiglusz, Piotr Kuropka, Jan Kuryszko, Agata Thannhauser, Lukasz Szleszkowski, Piotr Marian Wojtulek, Daniel Solinski, Piotr Dziegiel

**Affiliations:** 1 Department of Human Morphology and Embryology, Division of Normal Anatomy, Wroclaw Medical University, ul. Chalubinskiego 6a, 50-368 Wroclaw, Poland; 2 Department of Human Morphology and Embryology, Division of Ultrastructure Research, Wroclaw Medical University, ul. Chalubinskiego 6a, 50-368 Wroclaw, Poland; 3 Department of Human Morphology and Embryology, Division of Histology and Embryology, Wroclaw Medical University, ul. Chalubinskiego 6a, 50-368 Wroclaw, Poland; 4 Department of Anthropology, Institute of Immunology and Experimental Therapy, Polish Academy of Sciences in Wroclaw, ul. Weigla 12, 53-114 Wroclaw, Poland; 5 Institute of Low Temperature and Structure Research, Polish Academy of Sciences, ul. Okolna 2, 50-422 Wroclaw, Poland; 6 Centre for Advanced Materials and Smart Structures, Polish Academy of Sciences, ul. Okolna 2, 50-422 Wroclaw, Poland; 7 Department of Histology, Wroclaw University of Environmental and Life Sciences, ul. Norwida 25, 50-375 Wroclaw, Poland; 8 Department of Forensic Medicine, Wroclaw Medical University, ul. Mikulicza-Radeckiego 4, 50-345 Wroclaw, Poland; 9 Institute of Geological Sciences, University of Wroclaw, Pl. Borna 9, 50-205 Wroclaw, Poland

**Keywords:** syphilis, mercury, treponematoses, bioarchaeology, paleopathology

## Abstract

Venereal syphilis is a sexually transmitted disease caused by Treponema pallidum – Gram-negative, slowly growing bacteria. The spread of the disease in the Old World was due to increased birth rate, urban population growth, migration and lack of knowledge concerning the epidemiology. In the past, the treatment was mainly symptomatic and included application of mercury compounds. The goal of the study was to present the case of advanced venereal syphilis found in early modern (16th–18thc) graveyard localized in Wroclaw, Poland. The object of the study is a cranium of a male whose age at death has been estimated to be over 55. In order to observe the morphological and paleopathological characteristics of the examined material, anthropometrics, computed tomography, spectrometry and microscopic methods were incorporated. Microscopic analysis revealed the presence of the extensive inflammatory lesions. Analyses indicate tertiary stage of venereal syphilis as the most probable cause of the observed lesions. Concentration of arsenic (16.17±0.58 μg/g) in examined bone samples was about hundred times bigger than average arsenic concentration in bones reported in other studies. Advanced stage of observed lesions along with high arsenic level may suggest long-lasting palliative care and usage of arsenic compound in therapeutic treatment of this chronic disease.

## 1 Introduction

Venereal syphilis has been given many names over five centuries and they have been usually connected with the history of its spread (e.g., lues, French disease, Hungarian disease, or Neapolitan disease) [1]. The disease has been recognized for hundreds of years from South America to the Far East [2, 3, 4, 5, 6]. Among researchers, there are different opinions as for geographical regions of the primary outbreaks as well as paths of the disease spread. There is also no unanimity as whether syphilis originated from Europe or North or South of America. Some authors claim to have found evidence for syphilis in Europe even before Columbus discoveries [7, 8, 9], although other authors present just opposite arguments [10,11]. Genetic analyses of the sequenced genomes of modern *Treponema pallidum* strains indicate a common ancestor after the fifteenth century, within the early modern era [12]. Nevertheless, it had become a pandemic disease by the end of the 15^th^ century [13,14].

In Poland, the first well documented syphilis case was recorded in historical sources two years after Columbus’ return from his first expedition [15]. However, there is a study indicating a probable case of syphilis dating from the 14^th^ century from Wroclaw, Poland [16, 17, 18].

Venereal syphilis is a disease caused by *Treponema pallidum subsp. pallidum* ^–^ Gram-negative bacteria which is slowly growing, usually 6^–^15 μm long and 0.1^–^0.2 μm in diameter. It reveals small tolerance for extracorporeal conditions. There are no methods to grow the *T. pallidum* bacterial culture for clinical purposes [19]. One can get infected with venereal syphilis only by direct contact with infected tissue (usually as a result of sexual intercourse or contact with exudative lesions). Due to bacteria slow growth, it takes 2-3 weeks pass for the infection to develop the first symptoms. The disease is manifested with painless, generally solitary and indurated ulcerative lesions [19]. Besides venereal syphilis, *Treponema* bacteria are also responsible for other diseases, such us non-venereal/endemic syphilis (caused by *T. p. endemicum*), yaws (*T. p. pertenue)* and pinta (*T. caretum*). All these four diseases are defined as treponematoses. However, only venereal syphilis has been widely spread in Europe; whereas pinta remains restricted to Central and South America, yaws – to tropical regions and endemic syphilis – to Middle East mainly [20]. We can distinguish three stages of treponematoses. In venereal syphilis, primary symptoms (painless lesion called “chancre” located in the inoculation site, mostly on sex organs) disappear without medical treatment after 3 to 6 weeks with no visible scars [19,20]. Secondary stage occurs shortly after the onset of primary symptoms. It is manifested with diverse, painless rash ( 1-2 cm diameter), lesions on palms and soles, sore throat, fever, loss of appetite, headache and meningitis which take from a couple of weeks to several months. As the symptoms tend to take variant course, other diseases can be misdiagnosed instead of syphilis [19]. The third stage of the disease can develop in the period from a few years up to decades after infection and its most characteristic lesions include gummata (effect of chronic granulomatous processes) and non granulomatous inflammations [18,21,22]. Both types of lesions can affect bones as inflammation usually begins in periosteum or in the bone cortex and finally involves both structures as well as medullary cavity. They are characteristic for excessive osteosclerosis and soft tissue (skin, mucous membranes, nostrils) get ulcerated. Lesions resulting from tertiary syphilis are most often found in long bones, however, other bones, including skull, may be affected as well [22, 23, 24, 25].

In the past, venereal syphilis was called “the Great Imitator” for its diagnostic difficulties [25, 26, 27, 28]. Syphilis results in diverse inflammatory changes which are similar to the effects of other diseases like: myeloma, Langerhans cell histiocytosis, leprosy and other treponematoses [29].

Even at present, syphilis may evoke clinical problems being mistaken for other diseases (especially when a clinician is not acquainted with patient’s sexual history). Although the treatment may prove problematic in the case of antibiotic resistant strains or patient’s allergy to antibiotics [19], yet still it is curable. However, in the past ,there was no effective drug for this disease. Trials of syphilis treatment in medieval and early modern period concentrated mainly on “cleansing” the organism with the use of diaphoresis or diuresis [9]. However, despite applied methods, they could not bring expected healing effects [30]. Mercury and its derivatives were the most common agents applied in these procedures and they were widely promoted by Paracelsus (1493-1541) [9]. Signs of chronic mercury treatment can be observed in elevated concentrations of this element in bone material harvested from archaeological sites [31,32].

Undoubtedly, the spread of venereal syphilis within the Old World was due to increased birthrate, urban population growth, migration and lack of knowledge about its epidemiology [3,33]. Gilewska-Dubis (2000), in her study of medieval Wroclaw, observed that syphilis was one of the most frequent causes of death among city inhabitants in the late 15^th^ century. In Poland, the oldest case attributed to venereal syphilis is a skull and fragments of limb bones harvested from the church of St. Giles (14^th^ century)[16]. However, some authors still remain skeptical about this report [34]. The first case of venereal syphilis in Poland was officially recorded in 1495, [18] whereas the treatment of veneral syphilis on a large scale began in 1528, in St. Roch Hospital in Krakow. Half of the facilities in that place were dedicated solely to syphilis treatment. During last five centuries, this disease has been mentioned in many Polish written sources and described sign and symptoms imply venereal syphilis rather than other treponematoses [35]. This report, presents pathological evaluation of a of skull of an adult, which was subsequently classified as a possible case of venereal syphilis.

## 2 Material

**Fig. 1:**
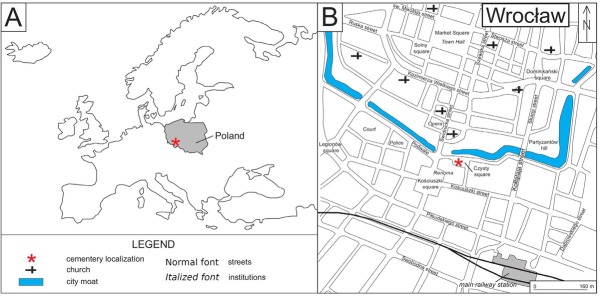
A: Wroclaw City/Poland B: Former Cemetery of our Saviour (Czysty Square/present)

The research was conducted on a skull obtained during excavations carried on in 2006-2007 on Pl. Czysty (Czysty Square) in Wroclaw (Poland), former Cemetery of Our Saviour (Fig. 1). Excavations were thoroughly described elsewhere [36,37].

In total, 1426 burials including 103 secondary interments were explored. The burials were often multiple, with visible displacements, disturbances and loose bones [36,37]. However, no sign of intentional looting or desecration were observed. The cemetery was used to bury inhabitants of surrounding villages and socially excluded people (such as suicides and convicts).

The analyzed skull (marked after the exploration of the grave as object No. 2000) was a isolated element without a sepulchral context or postcranial skeleton within grave No. 942, bearing the traces of grave reutilization [36].

All remains harvested from the cemetery were dated on the basis of information contained in parish chronicles and artifacts, i.e. coins or belt components. However, it is obvious that precise dating with coins in this case is rather inconclusive as in the graves, coins 100 years older than the cemetery were found. There were no Prussian coins found in findings which implies that the cemetery functioned at the latest to the first half of the 18^th^ century and it remains consistent with written historical sources [37,38]. The examined skull is be assessed as originating from the late 16^th^ to the mid 18^th^ century [36], which overlaps the whole period of the cemetery existence. The material consisted of skull without mandible, with ovoid shape in *norma verticalis*. Preliminary macroscopic examination revealed *antemortem* teeth loss and the presence of extensive osteolytic lesions involving the parietal bone cortex and about 60% of the frontal bone. It was the most damaged *antemortem* skull in the whole Cemetery of Our Saviour material.

## 3 Methods

### 3.1 Sex and age at death evaluation

The following macroscopic characteristics of skull were observed to define the sex of the individual: nuchal crest, mastoid process, supraorbital margin and prominence of glabella, [39,40]. The evaluation of sex was supported by analysis of skull morphometrics. The measurements used were g-op (glabella-ophistocranion), eu-eu (eurion-eurion), ba-b (basion-bregma), zy-zy (zygion-zygion), mf-ek (maxillofromtale-ektokonchion), sbk-spa (subconchion-supraconchion), apt-apt (aperthion-aperthion). Measurement listed were compared with mean values obtained for males and females from the cemetery.

Skull morphological features were examined with the use of measurement techniques established by Martin and Saller [41,42] with spreading caliper and sliding caliper and then compared with other skulls from Cemetery of Our Saviour [37]. Due to lack of postcranial skeleton, the age at death of the individual was assessed basing on cranial sutures obliteration[40], examination of histological slides as well as according to Kerley’s observations (1965) and finally, interstitial lamellae (remnants of former osteons, abundant in bones of old individuals) and the amount of unremodelled bone and the number of nonosteonal vascular canals (which are typical for bones of younger individuals) were evaluated.

### 3.2 Macroscopic evaluation of bone lesions

Assessment was performed with descriptive techniques including comparative data on the extent and shape of bone tissue defects [44,45].

Differential macroscopic analysis of bone lesions and osteolytic lesions were also performed in accordance with the differentiation criteria described by Ortner [22] and Putschar [46].

### 3.3 Computed tomography

Analysis of neurocranium and facial skeleton was made with the use of dual source computed tomography (DSCT) SIEMENS Somatom AS+; 80kv i 120kV/20 mAs; scanned with 1 mm layers.

### 3.4 Histological slides

Samples for histological analysis were harvested from the frontal bone in the Institute of Geological Sciences, University of Wroclaw with the use of a diamond saw.

Two methods were incorporated to elicit histological slides. The first sample was used to create a histological thick section with polisher-grinder and finally affixed to the glass. The second sample was fixed overnight in 4% formaldehyde which was followed by decalcification in EDTA for two weeks, and then for a month in sodium citrate and formic acid solutions. Decalcified sample was then used to prepare thin histological sections, which were stained with hematoxylin and eosin (H&E) with standard protocol [44].

The images were processed using Nikon 80i Eclipse with a UV-2A filter with a CCD Nikon camera (Nikon, Tokyo, Japan).

### 3.5 Electron Microscopy

Two 150 μm thick sections were prepared in the Institute of Geological Sciences at the University of Wroclaw for electron microprobe investigations. The major element composition of minerals was analyzed by electron microprobe (Cameca SX-100 at the Faculty of Geology, University of Warsaw, Poland) with energy dispersive X-ray spectroscopy (EDS). This method provides information on elemental and mineral composition of the histological section.

### 3.6 Analysis of elemental composition

Analysis was carried on 3 samples harvested from the left side of the frontal bone, 0,45 g each (powdered in agate mill) as well as from samples of dirt adjacent to the skull. The weighed amount of samples was etched in 2 ml of concentrated nitric acid, evaporated and dissolved in 10 ml.

The analysis was measured with inductively coupled plasma optical emission spectrometry (ICP-OES; Agilent 720,Wroclaw, Poland). Analytical lines: As: 194 nm, Hg: 273 nm and Pb: 220 nm.

## 4 Results

### 4.1 Sex and age at death evaluation

Diagnostic characteristics of the skull such as strongly defined glabella, massive superciliary arch, thickened supra-orbital margin and pronounced inferior nuchal line as well as external occipital protuberance indicate male sex of the individual.

**Fig. 2:**
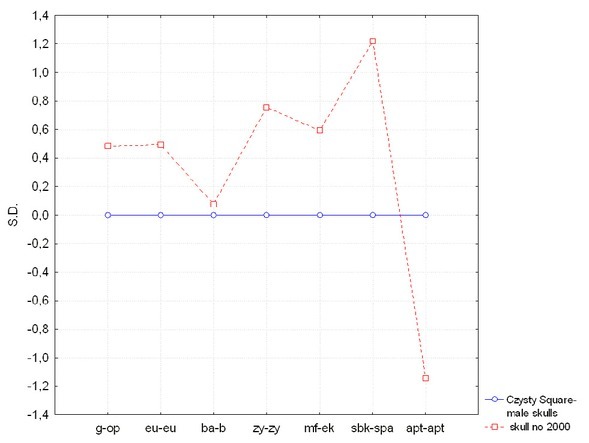
Profile presenting normalized values of differences between some measurements of the skull from grave no. 2000 and mean values of the assessment of male skulls from Czysty Square in Wroclaw. The anthropometric parameters do not differ significantly (differences do not exceed 1.96 S.D.) as compared to average values of measurements of male skulls from Czysty Square in Wroclaw, which suggest that the skull belonged to the male individual.

The skull was assessed as short and low in its neurocranial part (*brachycranius* and *tapeinocranius*), with a medium-height orbit (*mesoconch*). Detailed anthropometric parameters are presented in Figure 2. The profile presents normalized values of differences between certain measurements of the skull and mean values of the assessment for male skulls from Czysty Square in Wroclaw. There are no statistically significant disparities as they do not exceed 1.96 SD.

Morphological and histological characteristics of the skull were used to estimate the biological age as over 55 years. Assessment was based on the course and obliteration of sutures, i.e. sagittal and lambdoid ones. Histological assessment (suggesting similar age) was based on relatively high amount of fragments of remodeled osteons and low amount of non-osteal vascularization Numerous interstitial lamellae, as the remains of earlier generations of lamellae postponed from the periosteal and endosteal side and osteons in the middle part of the frontal bone were detected. They confirmed the observation that the skull belonged to an older adult [47]. More accurate assessment of age was impossible, as the postcranial skeleton was missing.

### 4.2 Macroscopic evaluation of bone lesions

**Fig. 3:**
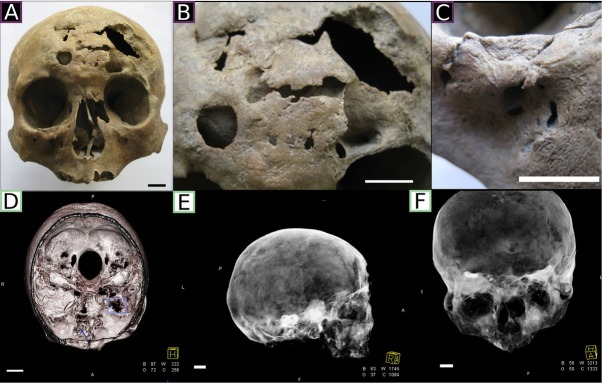
The skull 2000. A: Frontal view. B: Highly damaged frontal bone. C: Fracture in the right zygomaticomaxillary suture. D: Cross-horizontal-section of the skull in CT. Arrow and square indicate the inflammation on the base of the skull (anterior and middle cranial fossa) E: CT image of the right side of the skull. In the base of the vault lucencies are visible. F: CT image of the front of the skull. All damages and lesions of the skull have been thoroughly described in the results section. Scale bar=1cm.

Irregular malformation of 9 × 6.7 cm (Fig. 3A, B) was found within frontal bone left side squama. The lesions were configured as wide irregular bone defects extending to the anterior part of the frontal sinus on the left side of the frontal bone. The frontal bone in this region was markedly thinner as a result of extensive bone loss. The edges of this opening were thick, convex, and rounded, which was attributed to bone remodelling indicative of the sclerotic healing process. On the right side of the basal part of the frontal bone, there was a regular round lesion (1.0 cm in diameter) penetrating towards the interior of the frontal sinus. Below the left frontal tuber, there was an irregular defect, 3.5 × 1.3 cm, exposing the spongy tissue of the frontal bone and leading to the anterior cranial fossa.

Within the anterior nasal aperture, significant loss of nasal bone caused by the osteolytic process and a convex deformation near the frontonasal suture could be observed. On the left side of the nasal cavity, traces of osteolysis in the nasal conchae were visible, along with signs of osteolysis around the maxillary hiatus. The bony nasal septum showed traces of the healed fracture with vomer displacement towards the left side.

In the orbital plate of the ethmoid bone, an extensive oval bone defect was detected. The left frontal process of the maxilla was reduced and the right proved thinner. Deformation in the form of thickening of the lower edge of the right orbit could be also found, probably resulting from a healed Le Fort II fracture within the zygomaticomaxillary suture [48] (Fig. 3C).

The lesions penetrating the frontal bone and traces of osteolysis were confirmed by CT imaging. On the CT scans, the lesions are visible as lucencies and rarefactions in the cortical bone and diploë. Moreover, CT scans revealed the presence of an ongoing inflammatory process within the anterior cranial fossa and the left side of the middle cranial fossa (Fig. 3D, E).

### 4.3 Paleopathological differential diagnosis

In the macroscopic picture of the disease, the predominating signs are extensive osteolytic lesions, manifested as bone loss foci with sclerotic margins observed mainly on the frontal bone and within the nasal cavity structures. Such signs may result from granulomatous inflammation, thus other pathologies that share the mechanism of the infection should be taken into consideration in the differential diagnosis. One of granulomatous chronic infections is tuberculosis (caused by *Mycobacterium tuberculosis*). It can produce destructive lesions within different elements of the skeletal system [49]. However, even in the preantibiotic era, calvarial tuberculosis was extremely rare with the prevalence estimated at the level below 1% of all skeletal tuberculosis cases [22]. Leprosy was another mycobacterial disease considered as a possible cause of the pathological changes observed in the facial skeleton of the cranium No. 2000 for the fact that at the advanced stage of this disease, extensive damages within the nasal cavity (in the form of rhinomaxillary syndrome) could be observed. Leprosy is characteristic for resorption of the anterior nasal spine and the alveolar bone crest below as well as for the osteolysis within the upper alveolar arch. Bilateral changes in the shape of the piriform aperture, with remodeling of its edge [50,51] are another specific quality of this abnormality. In the examined case, we observed osteolysis only within the nasal cavity walls and nasal conchae with pronounced loss of the nasal bones. In the lower part of the piriform aperture, the anterior nasal spine was preserved, which suggested excluding leprosy as possible diagnosis.

The observation of multiple osteolytic lesions within the cranium No. 2000 from Czysty Square in Wroclaw was also the cause for concern for the possibility of the neoplastic etiology of the pathological changes. Among proliferative diseases which could have produced lesions similar to those observed in the case presented in our study, multiple myeloma (MM) and other tumors as well as Langerhans cell histiocytosis (LCH) were considered in differential diagnosis.

In the course of MM, numerous osteolytic foci, scallope-edged, round or oval in shape could be usually observed in the CT imaging. The most characteristic feature of those lesions was the extensive cortex loss without osteoblastic response. The most frequently observed clinical manifestation of MM in the cranium were numerous, well-delineated, lytic bone lesions and punched out lucencies which could be observed in CT imaging. [52,53]. Such changes were absent in the CT scans of the examined skull. Also, other tumors – metastatic or primary – were not likely to be included in this group. The margins of lesions caused by lytic metastases tend to be similar as for MM, however they may present osteoblastic reaction [54]. Blastic metastasis or benign tumor would result in osteoblastic lesions, absent on the skull.

Another option considered in differential diagnosis was LCH. The most frequent sign in the course of the disease is extensive loss within the flat bones (including skull bones). A description of the *clinical picture of LCH included numerous, round superficial defects and perforations of the skull vault without any visible signs of regenerative process* [55]. Lesions could be observed also in the basal part of sella turcica. This abnormality affects mainly juveniles and young adults (15-18 years old) [56]. In the examined cranium no analogy to this description was found.

Both localization and morphology of lesions can be considered as typical for late stage (tertiary) of treponematoses, i.e. venereal syphilis. In the course of venereal syphilis, the disease affects mainly these elements of the skeleton in which periosteum strictly adheres to the bone surface. They are mainly frontal and parietal bones of the neurocranium and proximal parts of the long bones shafts. The lesions within neurocranium characteristic for the tertiary syphilis are usually caused by gummata production, which subsequently stimulates the periosteum to inflammatory response. Gummata can derive from the subcutaneous tissue, bone, periosteum or muscles [57]. In the case of the cranial bones, gummata are usually situated on the frontal and parietal bones and they cause pathological changes called caries sicca. Caries sicca is a scar remaining after healing of superficial gummatous osteitis of calvaria [58]. This sequence of caries sicca formation begins from appearance of clustered pits followed by absorption of the cortical and cancellous bone leading to exposing wide regions of the cranial dura mater and ends with bone remodelling and scarring [24,59]. In the facial skeleton, the loss of nasal bones with destructive remodeling of piriform aperture and frontal processes of the maxilla can be observed [22]. Moreover, perforations in the thin-walled structures of the maxillary corpus and within the walls of the orbits are possible. The thinning of the lateral walls of the nasal cavity, palatine process and nasal septum may also occur. The shape of the nasal spine usually remains unchanged [24]. Pathological changes observed macroscopically as a complex of extensive osteolytic and hyperplastic lesions within the supraorbital region, as well as in the infraorbital and nasal part of the viscerocranium corresponded with the description of features characteristic for skeletal signs of tertiary stage of treponematose as the primary cause of the lesions observed in the cranium No 2000.

In conclusion, in respect of this paper, the signs of disease like perforating lesions with serpiginous cavitations and osteolysis, can be considered as the effects of a chronic inflammatory process associated with bone remodeling resulting in osteolytic complications. It was evidenced by the CT imaging as lucencies and rarefactions revealed in the cortical bone. It has already been mentioned that both macroscopic and CT images of the disease correspond with the traits characteristic for venereal syphilis as the most common treponematose [4,46,60]. Treponematoses have diverse course and many cases do not have characteristic pattern of skeletal lesions, which makes diagnosis quite challenging [60]. Also, the course may be modified by other infections accompanying treponemal syndromes, such as fungal or bacterial infections, which also may be destructive for nasal or palate tissue and cannot be excluded from diagnosis [54]. Although other treponematoses such as yaws or endemic syphilis cannot be clearly distinguished from venereal syphilis in the macroscopic evaluation, venereal syphilis is the most probable diagnosis. Yaws is usually acquired in childhood and is a tropical disease, uncommon in Central Europe, and endemic syphilis is usually most common in Middle East and Africa [22].

### 4.4 Histological examination

**Fig. 4:**
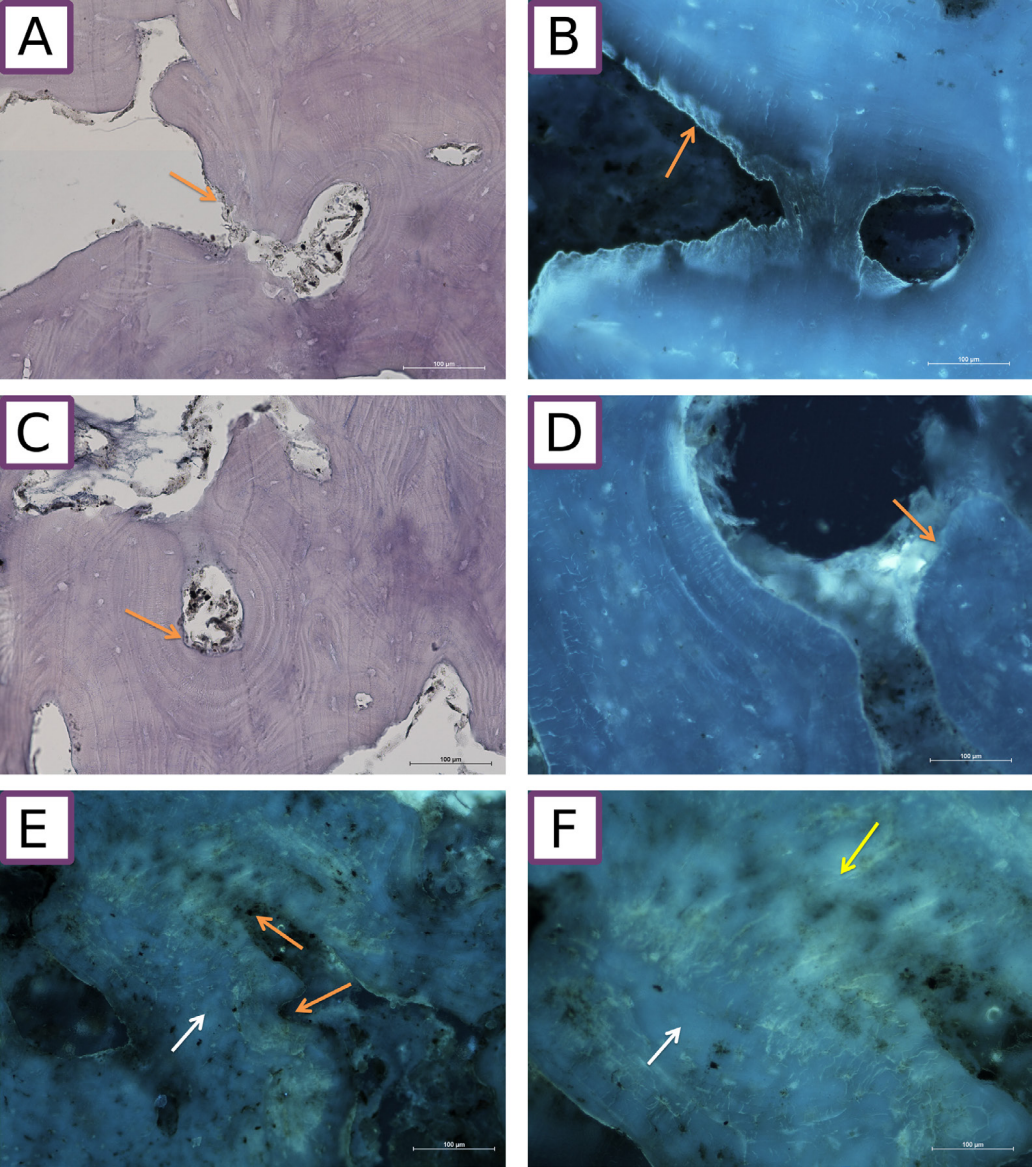
Histological microsections. A, C: Stained with H&E, light microscopy. B, D, E, F: Unstained, autofluorescence. There are areas of bone covered by several layers of bone lamellae and rough areas presenting osteolysis (orange arrows). Moreover, there is no trace of osteogenesis which should follow resorption. E,F: Diverse autofluorescence of different areas of bone matrix. Proper, blue (white arrow) and untypical, light-yellow (yellow arrow).

Histological examination revealed irregular bone matrix saturation with hydroxyapatite (Fig. 4). The inclusions of unspecified foreign compounds were found, visible as areas of different emission of excited light (Fig. 4F, yellow color). Different excisions of various intensity were observed in some lacunae and canaliculi. Because of the presence of minor impurities, there were lacunae emitting white-blue or yellow light or showing no excision. In some areas of the frontal bone, inclusions could be found extracellularly in the bone matrix. Bone tissue was mostly normal, yet there were areas with pronounced osteolysis with visible traces of regeneration (Fig 4A-E, orange arrows).

Analysis of slides stained with H&E confirmed observations made with fluorescent microscopy.

### 4.5 Electron microscopy

**Fig. 5:**
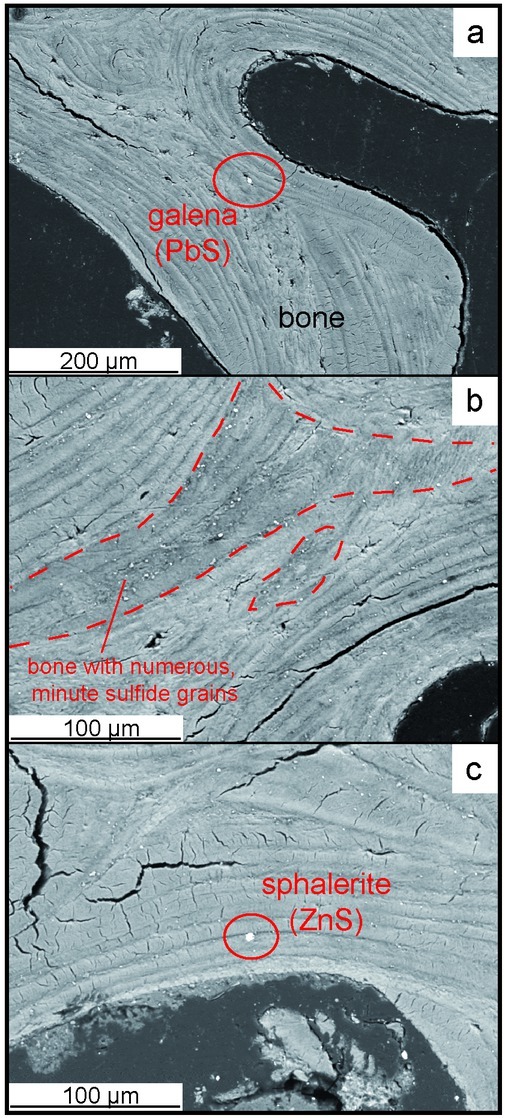
Back-scattered electron images of the bone from the Czysty Square: a - general view of the bone structure showing various generations of apatite, b- minute sulphide mineralization in the bone structure (surrounded by red line), c- a grain of sphalerite - ZnS (white) occurring between various kinds of apatite.

**Fig. 6:**
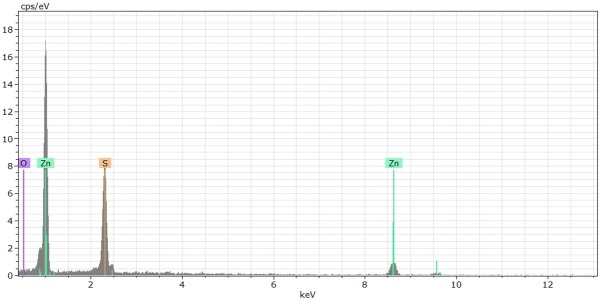
Representative EDS analysis of sulphide (sphalerite - ZnS) grains occurring within the bone showing the peaks of the Zn and S. Cps/eV – count per second electron‐volt, keV: kilo‐electron‐volt.

Electron EDS microscopy investigations of the samples showed that the bone consists mostly of apatite (Ca_5_(PO_4_)_3_). Locally numerous, minute (1-3 μm) sulphide inclusions (galena - PbS, sphalerite - ZnS and covellite - CuS) occured along boundaries between various generations of apatite (Fig. 5 and 6). As or Hg containing minerals were not detected.

### 4.6 Elemental composition evaluation

Results of analysis are presented in Table 1. Content of As, Hg and Pb is significantly higher in bone than in dirt samples. Cemetery of Our Saviour soil is mostly composed of sand, clay and loess formations. Its formation fits the lowland category, Fluvisols and Cambisol group. Brown and acid brown soils occur predominantly [61,62]. Obtained results are typical for soil in Wroclaw [62,63].

**Table 1:** Concentration of arsenic, mercury and lead in skull and adjacent dirt.

Element	Skull (μg/g)	Dirt (μg/g)
Arsenic (As)	16.17±0.58 μg/g	0.0954±0.006 μg/g
Mercury (Hg)	0.311±0.019 μg/g	0.0310±0.0010 μg/g
Lead (Pb)	44.02±0.71 μg/g	0.367±0.014 μg/g

## 5 Discussion

Skull No. 2000 belonged to a older adult male. It didn’t differ with its shape or anthropological parameters from average values calculated from all male skulls found in Cemetery of Our Saviour. The proportions corresponded with features of other male skulls from several Early Modern cemeteries in Wroclaw [64,65]

Macroscopic examinations indicated tertiary venereal syphilis. According to many researches, this disease is manifested (among others) with extensive osteolytic bone loss with an ongoing regeneration process [22,54,59]. In the case of the studied skull, the expanse of bone lesions of the frontal bone suggested bacterial spreading to dura mater and subdural structures up to nervous tissue [66]. Observed osteolytic loss and fistulas in the facial skeleton, especially in the center of frontal bone and within the nasal cavity along with remodeling of the surroundings of the frontonasal suture, indicated chronic disease [67], which might have had destructive influence on the nasociliary and facial nerve [68].

Rarefaction of the compact bone structure combined with endocranial perforating lesions visible in the CT image confirmed the destructive course of acquired syphilis. The osteolytic process penetration of the sinuses, anterior cranial fossa and areas to the left side of the middle cranial fossa was particularly visible.

Microscopic analysis (unstained thick sections and H&E stained sections) indicated extensive osteolysis, which was followed by bone regeneration. The region that was especially vulnerable were periosteal cell directly adjacent to skin. However, suppressed osteosynthesis was observed on the endosteal side. It could have been caused by arsenic, as it blocks proteins sulfhydryl groups, which leads to, among other things, cell cycle arrest in S phase and therefore to inhibition of bone regeneration [69,70].

Syphilis was treated in medieval and early modern times with mercury compounds, although the treatment was ineffective [9]. Such a therapy can result in the deposition of a considerable amount of this metal in the bones [32]. The concentration of mercury (Hg) in bone tissue differs according to the location of the sample. Increased Hg levels are observed in the most spongy structures, whereas in compact bone, levels are significantly lower [32]. There are no established standards for mercury concentration in bone structures. In some publications[31,71,72], the average concentration of Hg in bone tissue is approximately several dozen ng/g of dry mass. However, Rasmussen et al. (2008) found remains with no visible lesions (including syphilitic lesions) with a concentration of up to 300 ng/g of dry mass.

The results of chemical analysis revealed a moderately high Hg concentration (0.31 μg/g) in the examined bone fragments. It is unclear whether the subject was treated with mercury compounds. The concentration of this element in syphilitic patients treated with its compounds is usually significantly higher, even above 3 μg/g [31,73]. The presence of mercury in the skull may have been unrelated to the treatment of syphilis. In the Middle Ages and Early Modern period mercury was used as laxative as well as a drug for conjunctivitis, corneal irritation, psoriasis, eczema, tinea, skin lesions and others [74]. In addition, mercury was used as an ink and painting pigment, a gold treatment agent and a cosmetic ingredient [32].

The second heavy metal examined was arsenic, the concentration of which in frontal bone samples was 16.17±0.58 μg/g. However, the concentration of this element did not lead to formation of minerals detectable by EDS scanning microscopy, yet it exceeded significantly the values obtained by other researchers from bone material without long-lasting contact with arsenic compounds [72,75,76]. Diagenetic uptake of arsenic is unlikely, due to lack of this element is dirt samples. Its presence in bones can be explained in several ways.

Arsenic-containing medicines were already used in Antiquity. Hippocrates used aurapigment and realgar (As_2_S_3_ and As_2_S_2_, respectively) as ingredients in salves, whereas in ancient Rome, Galen used mentioned salts to treat ulcers [77]. In the Renaissance period, arsenic compounds were recommended by Paracelsus and William Whithering [77].

These early physicians used the antiseptic, antipyretic, cholagogic, diastolic, calming and tonic properties of arsenic compounds [78]. Although arsenic (As) was used in Chinese medicine [78], there is no information about common usage of As in medieval or Early Modern Europe. The first broadly used treatment of syphilis with arsenic compound was Fowler’s solution however, it was introduced in the second half of XVIII century, whereas the studied skull was older [79]. However, syphilis, called in the past “the Great Imitator” [29], was often misdiagnosed as a tumor, tuberculosis, bone inflammations or leprosy [26], which could be treated with arsenic compounds. In addition, arsenic was quite accessible in Lower Silesia, as arsenic mines were present nearby and could be widely used in medicine by the local community [80].

It is possible that As presence was not strictly connected with treatment, but rather with occupational activity. Arsenic was used as a component of paints or stained glass as well as for leather and wood preservation [78]. The processes associated with these products could have resulted in increased arsenic concentration in bones.

In Zloty Stok (about 80 km from Wroclaw), gold and arsenic mines have been located since 13th century [80]. Work in mining with arsenic salts could significantly increase the level of As in bone tissue [81]. However, this theory has a weak point, because the examined skull belonged to an elderly, sick man. Most likely, he would not have been in the condition for hard and physical work.

Acute and lethal arsenic poisoning significantly increases the concentration of arsenic in hair and soft tissues; it does not, however, leave significant traces in bone tissue [82]. Therefore, acute poisoning should be excluded in this case. The diagenetic intake of this element is also improbable, as the soil samples from the grave did not contain high amounts of As [83].

Lead concentration did not differ significantly from the mean values obtained in previous studies [72,84]. Minerals found by EDS microscopy (galena - PbS, sphalerite - ZnS and covellite – CuS) proved typical for bone tissue and did not indicate any pathological conditions [85] as did not forms of distinct biominerals in the bone structure, detectable by electron EDS microscopy.

## 6 Conclusions

The above results most likely indicate acquired syphilis treated with arsenic compounds or arsenic damage effects occupational activity. The issue of arsenic level in bones in Lower Silesia may be interesting for future research.

## Abbreviations

CT: computed tomography
LCH: Langerhans cell histiocytosis
H&E: hematoxylin and eosin stain
MM: multiple myeloma
